# Injuries, treatment, and impairment caused by different types of fireworks; results of a 10 year multicenter retrospective cohort study

**DOI:** 10.1186/s13049-020-00811-z

**Published:** 2021-01-07

**Authors:** Daan T. Van Yperen, Esther M. M. Van Lieshout, J. Niels Dijkshoorn, Cornelis H. Van der Vlies, Michael H. J. Verhofstad

**Affiliations:** 1 Trauma Research Unit Department of Surgery, Erasmus MC, University Medical Center Rotterdam, P.O. Box 2040, 3000 CA Rotterdam, The Netherlands; 2 Burn Center, Maasstad Hospital, P.O. Box 9100, 3079 DZ Rotterdam, The Netherlands

**Keywords:** Burns, Epidemiology, Fireworks, Impairment, Injury, Trauma

## Abstract

**Objectives:**

The primary aim of this study was to evaluate the number of patients reported to a hospital with injuries from consumer fireworks in the months December–January in the past 10 years, and to describe the association between the type of fireworks, injury pattern, treatment, and permanent impairment.

**Methods:**

A multicenter, retrospective, observational case series. Patients were selected from two hospitals in the Southwest Netherlands: a level 1 trauma center and a specialized burn center. All patients with any fireworks-related injuries treated between December 1 and January 31, during 2007 (December) to 2017 (January), were eligible for participation. The primary outcome was the number of patients with any type of injury caused by fireworks. The secondary outcome measures were patient and injury characteristics, treatment details, and whole person impairment (WPI). The percentage WPI expresses a patient’s degree of permanent impairments as a result of fireworks-related injuries.

**Results:**

Of the 297 eligible patients, 272 patients were included. From 2007 to 2017, between 21 and 40 patients were treated, and no clear increase or decrease was observed in the number of patients and in the number of patients per type of fireworks. Explosive fireworks mainly caused upper extremity (*N* = 65; 68%) injuries, while rockets (*N* = 24; 41%) and aerials (*N* = 7; 41%) mainly affected the head/neck. Decorative fireworks predominantly resulted in burns (*N* = 82; 68%), and explosive fireworks in soft tissue lacerations (*N* = 24; 25%), fractures (*N* = 16; 17%), and amputations (*N* = 14; 15%). Patients injured by explosive and homemade fireworks were most often admitted to a hospital (respectively *N* = 24; 36% and *N* = 12; 80%), and resulted in the highest proportion undergoing surgical procedures (respectively *N* = 22; 33% and *N* = 7; 47%). WPI found in this study was between 0 to 95%, with a median of 0%. In 34 (14%) patients, the injuries resulted in a WPI of ≥1%, mostly as a result of explosive fireworks (*N* = 18; 53%).

**Conclusion:**

This study found no increase or decrease in the number of patients treated in two specialized hospitals. Explosive and homemade fireworks could be considered as most dangerous, as they result into the most hospital admissions, surgical procedures, and into the most injuries with permanent impairment as a result.

## Introduction

Worldwide fireworks are used to invigorate joyful events, such as New Year’s Eve. However, despite their spectacular visible and audible effects, they can cause severe injuries, such as burns, eye injuries, and amputations [1–3]. These injuries can have devastating effects, with life-long consequences as a result. This does not only result in extra medical costs due to physical damage, but also in additional costs as a consequence of vandalism and the use of emergency services. These issues have stimulated a long-lasting public and political debate over the banning of consumer fireworks in the Netherlands.

Most fireworks-related injuries occur during the night of New Year’s Eve, because only then, the use of consumer fireworks is allowed in the Netherlands. Annually, between 400 and 1000 patients require hospital treatment. This number has been decreasing since 2012, to approximately 400 patients in 2019–2020 [4]. Most of them were young males, with in particular burns of the upper extremity or eye injuries [1, 3, 5–7].

The Dutch Safety Board – an independent advisory board for the national government – concluded in 2017 that New Year’s Eve is the most dangerous event in the Netherlands, and in order to improve the safeness of this event, they recommend to prohibit specific types fireworks for private use [8]. This board also emphasized the lack of scientific knowledge about the relation between the type of fireworks and the injuries it can cause. Such information is extremely important because it can guide policymakers in making decisions that improve the safe use of consumer fireworks.

Sandvall et al. reported that in patients treated at a level 1 Trauma/Burn Center, shells/mortars accounted for the greatest proportion of patients needing surgery [9]. This type of fireworks also caused the most injuries leading to permanent impairment. Among 130.000 pediatric patients, Billock et al. found that illegal/homemade fireworks resulted in the greatest proportion of hospital admissions [5]. Such data describing the effects of specific types of fireworks in the Netherlands are absent.

Because the number and severity of fireworks-related injuries varies widely from year to year [4], long term data are needed to provide a reliable overview of the consequences of fireworks. Therefore, the primary aim of this study was to evaluate the number of patients treated in a hospital with injuries from consumer fireworks in the months December–January in the past 10 years. The secondary aim was to describe the association between type of fireworks, injury pattern, treatment, and permanent impairment.

## Material and methods

### Study design & setting

This was a retrospective, multicenter, observational case series. Potential participants were selected from two tertiary referral hospitals in the Southwest Netherlands: a level 1 trauma center and a specialized burn center. This study was exempted by the Medical Research Ethics Committee Erasmus MC (Rotterdam, the Netherlands; registration number MEC-2018-1254).

### Participants

All patients (no age limit) with fireworks-related injuries, treated at one of the participating hospitals between December 1 and January 31 during the years 2007 (December) to 2017 (January), were eligible for participation. Patients were excluded if they died within the first 24 h due to other injuries and if patients’ medical records were incomplete regarding their injuries. Eligible patients (or parents/guardians) had to provide informed consent.

Potential participants were identified by searching the medical records on the terms “fireworks”, “bangers”, and “rockets”. They were screened for eligibility and were asked for informed consent by email or telephone. A reminder was sent after two and 4 weeks. Patients who refused participation were excluded. DTVY and JND screened patients for eligibility, obtained informed consent, and collected data by reviewing the patient’s medical records.

### Sample size calculation

A formal sample size calculation for this observational study was not constructive because of the descriptive and non-comparative study design. The Dutch Consumer Safety Institute – an institute responsible for public accident and injury prevention – mentioned 574 patients treated with fireworks-related injuries in 2014–2015 in the Netherlands [10]. With 2 months for inclusion and participation of two tertiary referral centers for advanced trauma care and burn injuries, approximately 200 patients in 10 years were estimated.

### Data collection

The primary outcome was the number of patients with any type of injury caused by fireworks. The number of injuries, including location and type (e.g., burns, soft tissue damage, or eye injury) were recorded. Burns were categorized as superficial, partial thickness (dermal), full thickness (subdermal), and mixed depth (partial and full thickness). Eye injury was categorized based upon the anatomical region affected, and soft tissue damage in superficial and deep lacerations. Amputations of digits were scored as full or partial. An amputation above the metacarpal phalangeal joint was considered as a partial amputation.

The secondary outcome measures were patient and injury characteristics, treatment details, and the percentage whole person impairment (WPI). Patient characteristics included age and gender. Accident information included the role of the patient (operator or bystander), the type of fireworks (explosive, decorative, calcium carbide, or homemade), and whether the fireworks was legal or illegal. Details regarding the definition of the different types of fireworks are provided in Table 1. Regarding treatment characteristics the hospital and intensive care unit (ICU) admission rates and length of stay were noted. Furthermore, the number and type of surgical interventions and the need for rehabilitation (physical, hand, and occupational therapist) was registered. For each injury, the percentage WPI was registered, according to the American Medical Association’s Guides to the Evaluation of Permanent Impairment [11]. For example: a complete hand amputation accounted for 56% WPI and a blind eye for 25%. In patients with more than one injury a combined WPI percentage was determined according to the guideline.

**Table 1:** Definition of different types of fireworks

Type of fireworks	Definition	Examples
Explosive fireworks	Fireworks primarily designed to explode and generate a large amount of noise (a bang). It hardly generates a visual effect. Often called firecrackers.	M-80’s, black cats, canon crackers, cobra’s
Decorative fireworks	Fireworks primarily designed to generate a visual effect, such as colorful flames.	
Rockets	A tube-like device, usually attached to a wooden stick, designed to propel itself into the air.	Bottle rockets, sky rockets, missiles
Aerials	A device designed to shoot flaming balls into the air.	Cakes, mortars,
Others	Small fireworks, mostly designed to be held in the hand or to be used on the ground. Only generates a flame and does not explode.	Sparkles, spinners, fountains
Carbide	Calcium carbide is a chemical compound that forms a highly flammable gas in reaction with water. When ignited it will explode. It is a tradition to use carbide to shoot objects from old milk cans.	
Homemade	Fireworks that were homemade or existing fireworks that was in any way altered. This type of fireworks was considered as illegal.	Pipe bombs

### Statistical analysis

Statistical analyses were performed using the Statistical Package for the Social Sciences version 25 (SPSS, Chicago, Ill., USA). Data were reported following the ‘Strengthening the Reporting of Observational studies in Epidemiology’ (STROBE) guidelines.

Normality of continuous data was tested with the Shapiro-Wilk test. This showed that all data deviated from the standard normal distribution. Missing values were not replaced by imputation.

Descriptive analyses were performed in order to report the data for the entire population as well as for the main types of fireworks. For continuous data, median and quartiles were reported. For categorical data, number and frequencies were reported. No statistical comparison was made between the types of fireworks.

## Results

### Participants

**Fig. 1:**
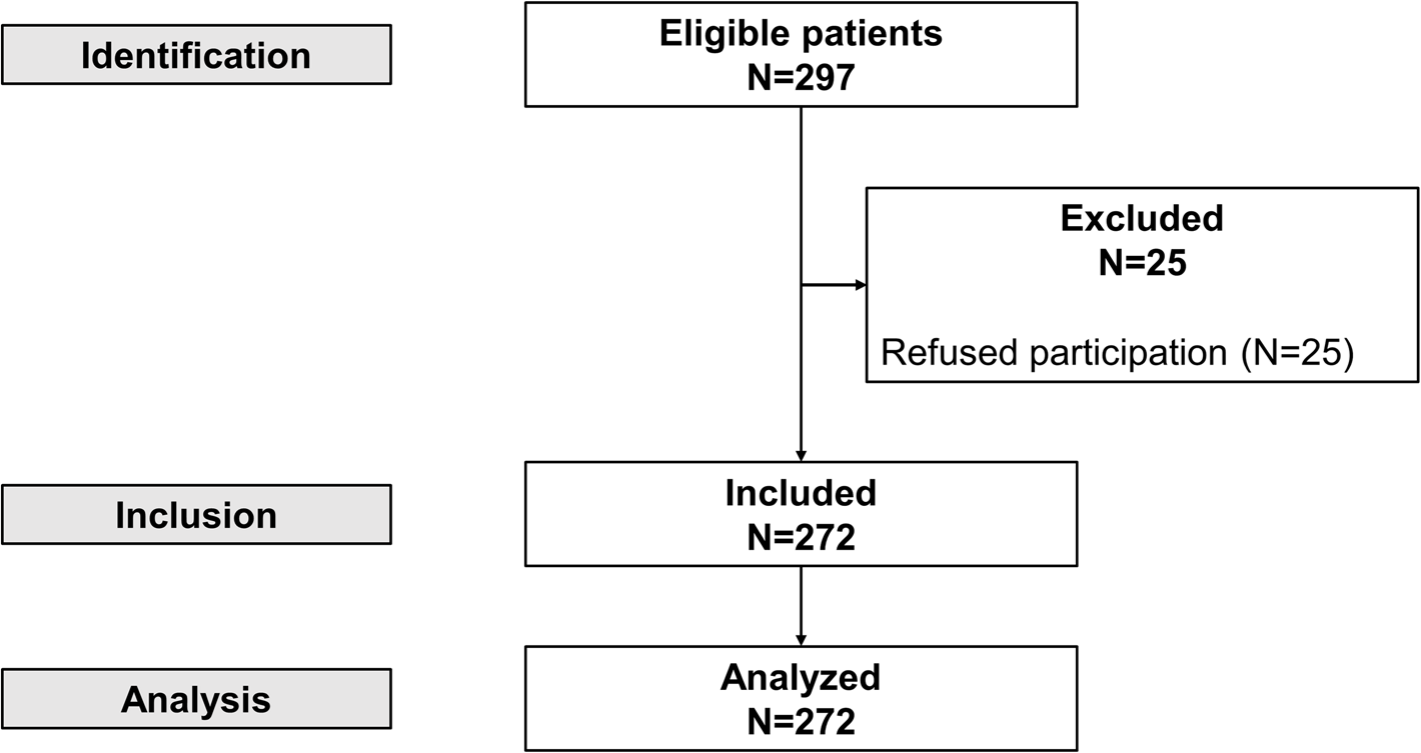
Study flow chart

Of the 297 patients who met the eligibility criteria, 25 patients were excluded because they refused to participate. A total of 272 patients were included in this study (Fig. 1). The median duration of clinical follow-up was 13 (P_25_-P_75_ 3–87) days.

**Fig. 2:**
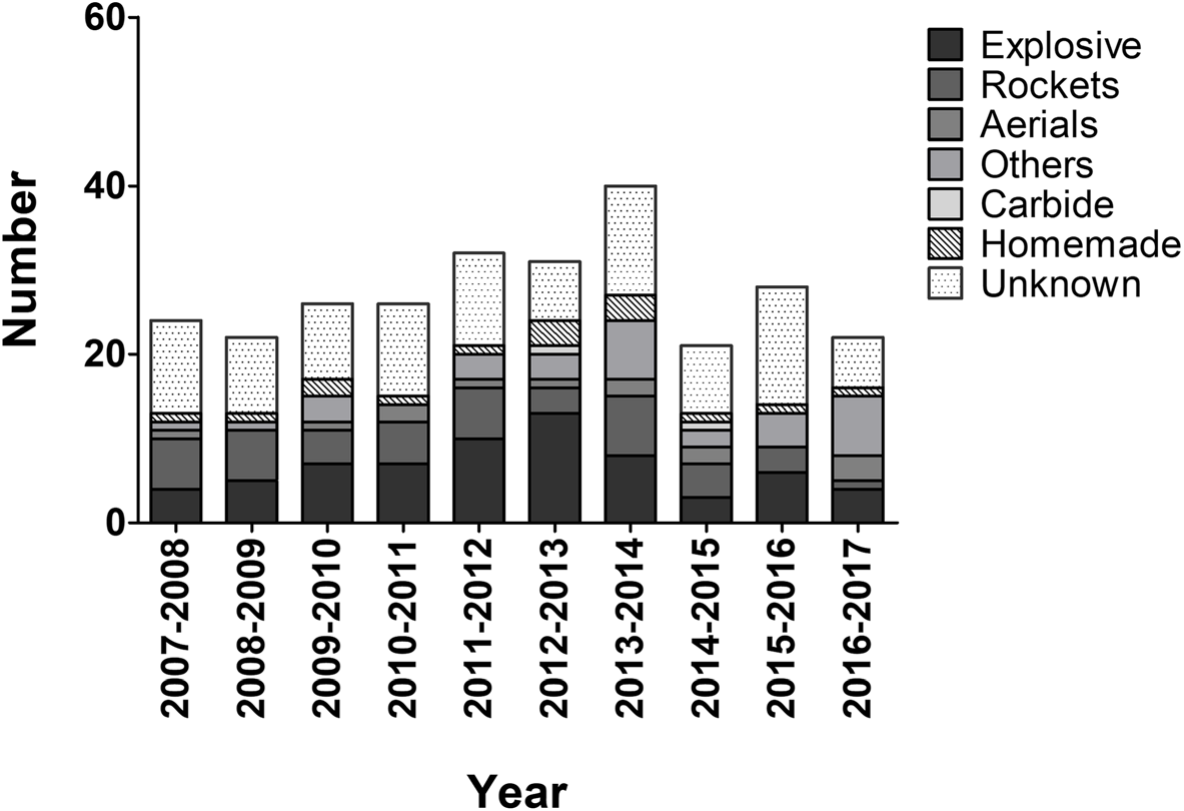
Number of patients treated per year. The number of patients with injuries from fireworks treated annually in December and January, per type of fireworks

The number of patients treated annually is shown in Fig. 2. This figure shows information for the entire group and for the different types of fireworks. From 2007 to 2017, between 21 and 40 patients were treated annually. The total number of patients treated increased from 24 in 2007 to 40 patients in 2014, and decreased again to 22 patients in 2017. From 2010 to 2014, explosive fireworks accounted for the greatest proportion of injuries. During this period, between 20% (*N* = 8) and 42% (*N* = 13) of patients had injuries induced by explosive fireworks. From 2014 this number decreased to levels comparable with other types of fireworks. The number of patients injured by rockets decreased over the last 4 years, from seven in 2014 to one in 2017. No clear increase or decrease was observed in the total number of patients treated and in the number of patients per type of fireworks.

### Patient characteristics

Information on patient and fireworks characteristics are presented in Table 2. For the entire group, the median age was 18 (P_25_-P_75_ 13–30) years. A total of 114 (42%) patients were children younger than 16 years old. The majority of patients (*N* = 227; 84%) were males and nearly half of all patients (*N* = 87; 40%) were bystanders. More than half of the patients (*N* = 50; 60%) were injured by legal fireworks.

**Table 2:** Patient and fireworks characteristics per type of fireworks

	All (***N*** = 272)		Explosive (***N =*** 67)		Decorative (***N =*** 89)						Carbide (***N =*** 2)		Homemade (***N =*** 15)		Unknown (***N*** = 99)	
						Rockets (***N =*** 45)		Aerials (***N =*** 13)		Others (***N =*** 31)						
	**N**^a^		**N**^a^		**N**^a^		**N**^a^		**N**^a^		**N**^a^		**N**^a^		**N**^a^	
Age (years)	272	18 (13–30)	45	16 (13–24)	45	21 (12–33)	13	29 (23–34)	31	13 (9–35)	2	21 (18–24)	15	19 (14–23)	99	20 (12–35)
Children	272	114 (42)	45	32 (48%)	45	17 (38%)	13	0 (0%)	31	18 (58%)	2	0 (0%)	15	7 (47%)	99	40 (40%)
Male	272	227 (84%)	45	61 (91%)	45	29 (64%)	13	12 (92%)	31	29 (94%)	2	2 (100%)	15	15 (100%)	99	79 (80%)
Bystander	217	87 (40%)	35	17 (27%)	35	27 (77%)	11	4 (36%)	30	11 (37%)	2	0 (0%)	13	1 (8%)	99	35 (57%)
Legal firework	83	50 (60%)	9	12 (40%)	9	9 (100%)	5	3 (60%)	24	24 (100%)	2	2 (100%)	13	0 (0%)	99	N.A.

Data are shown as median (P_25_-P_75_) or as N (%)

*N.A.* Not applicable

^a^This represents the number of patients for whom data were available

The majority of injuries were caused by decorative fireworks (*N* = 89; 51%), in particular rockets (*N* = 45; 50%), followed by explosive fireworks (*N* = 67; 39%). In 99 (25%) patients the type of fireworks was unknown. The median age differed widely per type of fireworks, from 13 years in the group ‘others’ to 29 years in the group of aerials. In all subgroups, males accounted for more than 80% of the patients, except for the rockets subgroup (*N* = 29; 64%). Rockets had the highest percentage of bystanders (*N* = 27; 77%); all other groups had less than 39% bystanders.

### Injury characteristics

In 272 patients a total of 395 injuries were reported. Most injuries were located to the upper extremity (*N* = 177; 45%), followed by the head/neck (*N* = 101; 26%), and the eyes (*N* = 49; 12%; Table 3). Explosive fireworks mainly resulted in upper extremity (*N* = 65; 68%) injuries, while rockets (*N* = 24; 41%) and aerials (*N* = 7; 41%) mainly affected the head/neck region.

**Table 3:** Location and type of injury per type of fireworks

	All (***N =*** 395)		Explosive (***N*** = 95)		Decorative (***N =*** 125)						Carbide (***N =*** 5)		Homemade (***N =*** 31)		Unknown (***N =*** 139)	
					Rockets (***N =*** 58)		Aerials (***N =*** 17)		Others (***N =*** 50)							
	**N**^a^		**N**^a^		**N**^a^		**N**^a^		**N**^a^		**N**^a^		**N**^a^		**N**^a^	
**Injury location**																
Head/neck	395	101 (26%)	95	7 (7%)	58	24 (41%)	17	7 (41%)	50	13 (26%)	5	2 (40%)	31	10 (32%)	139	38 (27%)
Eye	395	49 (12%)	95	8 (8%)	58	12 (21%)	17	3 (18%)	50	3 (6%)	5	2 (40%)	31	3 (10%)	139	18 (13%)
Ear	395	13 (3%)	95	8 (9%)	58	0 (0%)	17	0 (0%)	50	3 (6%)	5	1 (20%)	31	0 (0%)	139	1 (1%)
Upper extremity	395	177 (45%)	95	65 (68%)	58	13 (22%)	17	5 (29%)	50	24 (48%)	2	0 (0%)	31	14 (45%)	139	56 (40%)
Lower extremity	395	31 (8%)	95	5 (5%)	58	3 (5%)	17	0 (0%)	50	3 (6%)	5	0 (0%)	31	3 (10%)	139	17 (12%)
Trunk	395	24 (6%)	95	2 (2%)	58	6 (10%)	17	2 (12%)	50	4 (8%)	5	0 (0%)	31	1 (3%)	139	9 (7%)
**Type of injury**																
Burn	395	208 (53%)	95	20 (21%)	58	28 (48%)	17	12 (71%)	50	42 (84%)	5	2 (40%)	31	18 (58%)	139	86 (62%)
Superficial	208	13 (6%)	20	2 (10%)	28	2 (7%)	12	1 (8%)	42	6 (14%)	2	0 (0%)	18	0 (0%)	86	2 92%)
Partial thickness	208	162 (78%)	20	14 (70%)	28	21 (75%)	12	10 (83%)	42	29 (69%)	2	2 (100%)	18	15 (83%)	86	71 (83%)
Full thickness	208	24 (12%)	20	1 (5%)	28	3 (11%)	12	0 (0%)	42	6 (14%)	2	0 (0%)	18	3 (17%)	86	11 (13%)
Mixed	208	9 (4%)	20	3 (15%)	28	2 (7%)	12	1 (8%)	42	1 (2%)	2	0 (0%)	18	0 (0%)	86	2 (2%)
Eye injury	395	49 (12%)	95	8 (8%)	58	12 (21%)	17	3 (18%)	50	3 (6%)	5	2 (40%)	31	3 (10%)	139	18 (13%)
Ocular surface	46	36 (78%)	8	6 (75%)	12	7 (58%)	2	2 (100%)	3	3 (100%)	2	2 (100%)	3	2 (67%)	16	14 (88%)
Anterior chamber	46	2 (4%)	8	0 (0%)	12	0 (0%)	2	0 (0%)	3	0 (0%)	2	0 (0%)	3	0 (0%)	16	2 (13%)
Posterior segment	46	1 (2%)	8	0 (0%)	12	0 (0%)	2	0 (0%)	3	0 (0%)	2	0 (0%)	3	0 (0%)	16	1 (16%)
Adnexal	46	12 (26%)	8	0 (0%)	12	5 (42%)	2	0 (0%)	3	1 (33%)	2	2 (100%)	3	2 (67%)	16	2 (13%)
Fully destructed	46	6 (13%)	8	2 (25%)	12	2 (17%)	2	0 (0%)	3	0 (0%)	2	0 (0%)	3	1 (33%)	16	1 (6%)
Soft tissue laceration	395	67 (17%)	95	24 (25%)	58	13 (22%)	17	2 (12%)	50	1 (2%)	5	0 (0%)	31	3 (10%)	139	24 (17%)
Superficial	67	48 (73%)	24	21 (88%)	13	9 (69%)	2	1 (50%)	1	1 (100%)	0	0 (0%)	3	1 (33%)	139	15 (63%)
Deep	67	19 (28%)	24	3 (13%)	13	4 (31%)	2	1 (50%)	1	0 (0%)	0	0 (0%)	3	2 (67%)	139	9 (38%)
Fracture	395	31 (8%)	95	16 (17%)	58	4 (7%)	17	0 (0%)	50	1 (2%)	5	0 (0%)	31	4 (13%)	139	6 (4%)
Scull	31	1 (3%)	16	1 (6%)	4	0 (0%)	0	0 (0%)	1	0 (0%)	0	0 (0%)	4	0 (0%)	6	0 (0%)
Face	31	6 (19%)	16	1 (6%)	4	3 (75%)	0	0 (0%)	1	0 (0%)	0	0 (0%)	4	1 (25%)	6	1 (17%)
Wrist	31	1 (3%)	16	0 (0%)	4	0 (0%)	0	0 (0%)	1	0 (0%)	0	0 (0%)	4	1 (4%)	6	0 (0%)
Hand	31	7 (23%)	16	5 (31%)	4	0 (0%)	0	0 (0%)	1	1 (100%)	0	0 (0%)	4	1 (25%)	6	0 (0%)
Finger	31	19 (61%)	16	12 (75%)	4	0 (0%)	0	1 (25%)	1	0 (0%)	0	0 (0%)	4	2 (50%)	6	4 (67%)
Leg	31	1 (3%)	16	0 (0%)	4	0 (0%)	0	0 (0%)	1	0 (0%)	0	0 (0%)	4	0 (0%)	6	1 (3%)
Amputation	395	19 (5%)	95	14 (15%)	58	0 (0%)	17	0 (0%)	50	1 (3%)	5	0 (0%)	31	2 (7%)	139	2 (1%)
Vascular injury^a^	395	1 (0%)	95	1 (1%)	58	0 (0%)	17	0 (0%)	50	0 (0%)	5	0 (0%)	31	0 (0%)	139	0 (0%)
Neural injury^b^	395	1 (0%)	95	1 (1%)	58	0 (0%)	17	0 (0%)	50	0 (0%)	5	0 (0%)	31	0 (0%)	139	0 (0%)
Tendon injury^c^	395	1 (0%)	95	1 (1%)	58	0 (0%)	17	0 (0%)	50	0 (0%)	5	0 (0%)	31	0 (0%)	139	0 (0%)
Eardrum perforation	395	11 (3%)	95	8 (8%)	58	0 (0%)	17	0 (0%)	50	2 (4%)	5	1 (20%)	31	0 (0%)	139	0 (0%)
Contusion	395	5 (1%)	95	1 (1%)	58	1 (2%)	17	0 (0%)	50	0 (0%)	5	0 (0%)	31	0 (0%)	139	3 (2%)
Other^c^	395	2 (1%)	95	1 (1%)	58	0 (0%)	17	0 (0%)	50	0 (0%)	5	0 (0%)	31	1 (3%)	139	0 (0%)

Data are shown as N (%)

^a^This represents the number of injuries for which data were available

^a^Ulnar artery and common palmar digital artery

^b^Common digital palmar nerve

^c^Flexor digitorum superficialis & profundus

^d^Colon perforation and severe brain damage.Table 3 Treatment per type of fireworks

Burns were the most common injuries (*N* = 208; 53%), and they were mainly of partial thickness (*N* = 162; 78%). The second most common type of injuries were soft tissue lacerations (*N* = 67; 17%), of which most were superficial (*N* = 48; 73%), and the third most common type were eye injuries (*N* = 49; 12%). Six patients had bilateral eye injury. One child and five adults had a fully destructed eye and were considered legally blind. Thirty-one (8%) injuries were fractures, of which the finger (*N* = 19; 61%), hand (*N* = 7; 23%), and face (*N* = 6; 19%) were the most common locations. Furthermore, 19 (5%) injuries were amputations, which were all located to the hand or fingers. Two hands, 32 fingers (29 partial), and four thumbs (two partial) were amputated.

Decorative fireworks mostly resulted in burn injuries (*N* = 82; 68%), of which 60 (73%) injuries were of partial thickness. Explosive fireworks mostly caused soft tissue lacerations (*N* = 24; 25%), fractures (*N* = 16; 17%), and amputations (*N* = 14; 15%). Homemade fireworks mostly caused burns (*N* = 18; 58%). Eye injuries were mostly caused by rockets (*N* = 12; 24%) and explosive fireworks (*N* = 8; 16%).

### Treatment

In total 79 (29%) patients were admitted to a hospital for treatment, and 10 patients (4%) were admitted to the ICU (Table 4). The median hospital length of stay was 4 (P_25_-P_75_ 2–9) days. Fifty-one (19%) patients underwent surgery, of whom 22 (42%) underwent two or more procedures, with a maximum of 10. Furthermore, 57 (22%) patients received professional rehabilitation, of which most consulted a physical therapist (*N* = 29; 51%) or a hand therapist (*N* = 35; 61%).

**Table 4:** Treatment per type of fireworks

	All (***N =*** 272)		Explosive (***N =*** 67)		Decorative (***N =*** 89)						Carbide (***N =*** 2)		Homemade (***N =*** 15)		Unknown (***N =*** 99)	
					Rockets (***N =*** 45)		Aerials (***N =*** 13)		Others (***N =*** 31)							
	**N**^a^		**N**^a^		**N**^a^		**N**^a^		**N**^a^		**N**^a^		**N**^a^		**N**^a^	
**Treatment**																
Hospital admission	272	79 (29%)	67	24 (36%)	45	8 (18%)	13	3 (23%)	31	6 (19%)	2	2 (100%)	15	12 (80%)	99	24 (24%)
Length of stay (days)	79	4 (2–9)	24	5 (3–13)	8	1 (1–3)	3	1 (1–1)	6	9 (3–14)	2	1 (1–1)	12	1 (1–2)	24	3 (1–7)
ICU admission	272	10 (4%)	67	1 (2%)	45	3 (7%)	13	0 (0%)	31	1 (3%)	2	1 (50%)	15	2 (13%)	99	2 (2%)
Length of stay (days)	10	4 (2–7)	1	14 (14–14)	3	2 (2–5)	0	0 (0–0)	1	4 (4–4)	1	2 (2–2)	2	4 (2–5)	2	11 (4–17)
Operative treatment	272	51 (19%)	67	22 (33%)	45	6 (13%)	13	1 (8%)	31	3 (10%)	2	0 (0%)	15	7 (47%)	99	12 (12%)
≥ 2 surgery	51	22 (43)	22	11 (50%)	6	2 (33%)	1	1 (100%)	3	1 (33%)	0	0 (0%)	7	3 (43%)	12	4 (33%)
Rehabilitation needed	265	57 (22%)	66	24 (36%)	42	6 (14%)	12	1 (8%)	30	4 (13%)	2	1 (50%)	15	5 (33%)	98	16 (16%)
Physical therapy	57	29 (51%)	24	6 (25%)	6	4 (67%)	1	1 (100%)	4	3 (75%)	1	1 (100%)	5	4 (80%)	16	10 (63%)
Hand therapy	57	35 (61%)	24	22 (92%)	6	0 (100%)	1	1 (100%)	4	3 (75%)	1	0 (0%)	5	4 (80%)	16	5 (31%)
Occupational therapy	57	14 (25%)	24	3 (13%)	6	3 (50%)	1	0 (0%)	4	3 (75%)	1	0 (0%)	5	1 (20%)	16	4 (25%)

Data are shown as median (P_25_-P_75_) or as N (%)

^a^This represents the number of patients for whom data were available

*ICU* Intensive Care Unit

Patients with injuries induced by carbide and homemade fireworks were most often admitted to a hospital (respectively *N =* 2; 100% and *N* = 12; 80%), followed by patients injured by explosive fireworks (*N* = 24; 36%). Patients from the subgroup ‘others’ had the longest hospital length of stay; 9 (P_25_-P_75_ 3–18) days. Homemade (*N* = 7; 47%) and explosive fireworks (*N* = 22; 33%) resulted in the highest percentage of injuries that required surgical treatment.

### Whole person impairment (WPI)

In this study, no fatal casualties were observed. The WPI found in this study ranged from 0 to 95%. In 34 (14%) patients, the injuries resulted in a WPI of ≥1%; 18 (53%) patients used explosive fireworks, four (12%) homemade fireworks, four (12%) rockets, two (6%) ‘others’, and six (18%) the type of fireworks was unknown. In 217 (84%) patients the injuries did not result into permanent impairment and in 21 (8%) patients it was not possible to determine the WPI due to missing data. In all groups, the median WPI was 0% (P_25_-P_75_ 0–0).

One child sustained traumatic brain injury resulting from shrapnel from an illegal banger, resulting in 95% WPI. Another child lost a hand from a banger and one adult lost a hand from unknown fireworks, both leading to a WPI of 56%. Seventeen patients with one or more amputations of the upper extremity had a WPI between 2% for a single fingertip amputation, and to 46% for extensive hand trauma with multiple amputations of digits. In fourteen of these patients the injuries were induced by explosive fireworks. Six patients lost an eye and turned blind, resulting in a WPI of 25%. Blind eyes were caused by explosive fireworks (*N* = 2), rockets (*N =* 2), homemade fireworks (*N* = 1), and unknown fireworks (*N =* 1).

## Discussion

This study retrospectively investigated patients treated in two specialized hospitals for fireworks-related injuries over a 10 year period. The major finding was that the annual number of patients treated varied between 21 and 40 per year, and showed no increase or decrease over the past decade. Furthermore, injuries caused by explosive and homemade fireworks most often led to hospital admission, surgical treatment, and permanent impairment.

Similar to literature from the past two decades, the number of patients treated annually did not show an increase or decrease over the past years [1, 2, 5, 12–16]. On a nationwide level, the absolute number of patients treated in the Netherlands has decreased from 700 patients in 2014 to 385 in 2020 [4]. For years, policymakers have tried to reduce the number of fireworks-related injuries through informational and educational campaigns. A recently published before-after study by Homaie Rad et al. highlighted the effectiveness of such interventions, in terms of reduced disability-adjusted life years and burden of disease [17]. However, the effects of such campaigns have not yet been observed in the studies mentioned previously. Perhaps these campaigns were only effective on patients with minor injuries, who did not need hospital treatment. The preventive measures were not as effective as expected, while on the other hand, restrictive legislation has proven to be effective in reducing the number of fireworks-related injuries [18–20].

Only two previous studies described the type of fireworks in association to the injuries, treatment, and outcome. Comparing these studies was difficult because of differences in grouping the types of fireworks. A study from the United States investigated more than 130.000 pediatric patients and found that illegal/homemade fireworks accounted for the greatest proportion of hospital admissions [5]. However, they combined illegal firecrackers with homemade fireworks, whereas in this current study both legal and illegal firecrackers were combined in the explosive fireworks. A study from Sandvall et al. included patients from a level 1 trauma and burn center, and observed the greatest proportion of operations among the shells/mortars group, followed by homemade fireworks. These two groups also caused the greatest proportion of eye and hand injuries that led to permanent impairment. Both studies mentioned above show similarities with our study, and illustrate the dangers of homemade and (illegal) explosive fireworks. Homemade fireworks per definition are illegal and thus not allowed to be used privately, but not all explosive fireworks are illegal. This study made no distinction between legal and illegal fireworks because this was registered in only less than a third of patients.

This study also showed that in particular rockets were dangerous to bystanders. The proportion of bystanders affected by rockets was much higher than in other types of fireworks. In rockets not only the operator is at risk, but also the people nearby spectating. This has also been reported before [9]. Rockets are often fired from an empty bottle, and can easily trip or deviate and hit bystanders. The Dutch Safety Board in 2017 recommended to prohibit the private use of rockets, as well as the use of explosive fireworks, because these two types of fireworks most infringed the overall safety during New Year’s Eve [8]. In 2020, the Dutch government followed this advice and introduced a law that prohibits the private use of explosive fireworks and rockets. The effects of this intervention are yet to be observed.

To our knowledge, this study is among the few that calculated permanent impairment from fireworks-related injuries. Only Sandvall *et. al* did so and found a WPI between 1 and 77%, which is comparable to the current study [9]. A WPI between 0 and 95% was found in this current study. This wide range was mainly a result of the low number of patients in which permanent impairment was objectified, and due to the strong variety in injury pattern each year.

Most injuries leading to permanent impairment were caused by explosive and homemade fireworks. Fourteen out of 18 patients with permanent impairment from explosive fireworks had amputations. In this type of fireworks the blast in particular can easily result into amputations, which irrefutable leads to permanent damage. Decorative fireworks, on the other hand, mainly generates a flash instead of an explosion, and thus mostly causes superficial burns small in size [2, 16]. Although a substantial portion of patients from this study presumably have scars, this is not reflected by a the percentage whole person impairment since impairment from scars only arises when 10% of the total body surface is affected. Therefore, permanent impairment is much less likely to occur from decorative fireworks than from explosive fireworks. Nevertheless, the widespread range of the percentage of WPI found in this study, illustrates the potential and devastating effects of fireworks. Whether this impairment also results into a functional impairment has not been investigated extensively. Previous research found limited reduced quality of life and functional outcome 1 year after trauma the reported [3].

### Strengths and limitations

A strength of this study is that it provides a long-term overview of patients who were treated in tertiary referral centers that frequently treat patients with severe fireworks-related injuries. This information provides more insight into the number of patients treated over the past 10 years and the outcomes after multiple years. It also provides more information about the injuries caused by fireworks and describes the association between the type of fireworks and the injuries, treatment, and to what extent it led to permanent impairment. This will help policy makers in developing the best strategy to prevent firework-related injuries.

A limitation in this study could be that only two specialized hospitals were selected to participate in this study. The aim of this study was to focus on the severely injured patients, who are more likely to be treated at a specialized hospital. However, patients with severe eye injuries were often immediately transported to a nearby specialized eye hospital, which functions as a national referral center. Therefore these patients were not included in this study. This likely led to an under representation of the patients with severe eye injuries. Data of these patients treated at this specialized eye hospital were recently published [21]. Furthermore, because of the retrospective design of this study, some data were not registered in patients’ medical records, such as the type of fireworks. This requires caution in making statements about causality. Moreover, a potential weakness of this study is that functional impairment was calculated based on data from hospital records only. For amputations and ocular enucleations, it is clear that impairment will remain. But it is likely that the degree of impairment – also for those who scored 0% WPI now – would have been higher after a specific physical investigation for the purpose of this study. An underestimation due to the calculation based on short term hospital records is very well possible.

## Conclusion

The primary aim of this study was to evaluate the number of patients treated in a hospital with injuries from consumer fireworks in the months December–January in the past 10 years, and to describe the association between type of fireworks, injury pattern, treatment, and permanent impairment. No increase of decrease in the number of patients treated was found during the study period. This study found that explosive and homemade fireworks could be considered as most dangerous, as they are associated with the highest rates of admissions, surgical procedures, and patient with permanent impairment as a result of their injuries. Rockets in particular were dangerous to bystanders. Although most of the injuries did not result into permanent impairment, some patients suffered lifelong and severe disabilities.

The results of this study must contribute to the public and political debate, and help policymakers worldwide in tackling the problems arising from using consumer fireworks. Future research should focus on developing safer alternatives for fireworks, for example lasers, drones, and other non-fire generating devices.

## Data Availability

Anonymized data analyzed for the current study will be shared if a reasonable request is made by a qualified investigator to the corresponding author.

## Abbreviations

ICU: Intensive Care Unit
MEC: Medical Ethics Committee
WPI: Whole Person Impairment
